# Association of active immunotherapy with outcomes in cancer patients with COVID-19: a systematic review and meta-analysis

**DOI:** 10.18632/aging.203945

**Published:** 2022-03-10

**Authors:** Chang Cao, Xinyan Gan, Xiaolin Hu, Yonglin Su, Yu Zhang, Xingchen Peng

**Affiliations:** 1 State Key Laboratory of Oral Diseases, West China Hospital of Stomatology/Cancer Center, West China Hospital, Sichuan University, Chengdu 610041, Sichuan, P.R. China; 2 Department of Nursing, West China Hospital, Sichuan University, Chengdu 610041, Sichuan, P.R. China; 3 Department of Biotherapy, Cancer Center, West China Hospital, Sichuan University, Chengdu 610041, Sichuan, P.R. China; 4 Affiliated Hospital of Chengdu University, Chengdu 610041, Sichuan, P.R. China

**Keywords:** COVID-19, cancer, immunotherapy, safety, meta-analysis

## Abstract

Background: During the COVID-19 pandemic, there are growing concerns about the safety of administering immunotherapy in cancer patients with COVID-19. However, current clinical guidelines provided no clear recommendation.

Methods: Studies were searched and retrieved from electronic databases. The meta-analysis was performed by employing the generic inverse-variance method. A random-effects model was used to calculate the unadjusted odds ratios (ORs) and adjusted ORs with the corresponding 95% CIs.

Results: This meta-analysis included 20 articles with 6,042 cancer patients diagnosed with COVID-19. According to the univariate analysis, the acceptance of immunotherapy within 30 days before COVID-19 diagnosis did not increase the mortality of cancer patients (OR: 0.92; 95% CI: 0.68-1.25; *P*=0.61). Moreover, after adjusting for confounders, the adjusted OR for mortality was 0.51, with borderline significance (95% CI: 0.25-1.01; *P*=0.053). Similarly, the univariate analysis showed that the acceptance of immunotherapy within 30 days before COVID-19 diagnosis did not increase the risk of severe/critical disease in cancer patients (OR: 1.07; 95% CI: 0.78-1.47; *P*=0.66). No significant between-study heterogeneity was found in these analyses.

Conclusions: Accepting immunotherapy within 30 days before the diagnosis of COVID-19 was not significantly associated with a higher risk of mortality or severe/critical disease of infected cancer patients. Further prospectively designed studies with large sample sizes are required to evaluate the present results.

## INTRODUCTION

As of 17 September 2021, a total of 226,844,344 confirmed COVID-19 cases were reported globally, and 4,666,334 COVID-19-related deaths were reported [1]. Cancer is revealed to be independently associated with the risk of COVID-19 and is significantly related to an increased rate of severe disease and mortality [2]. SARS-CoV-2 has been shown to trigger cytokine storms, leading to the increment of the incidence of acute respiratory distress syndrome (ARDS). Thus, during the pandemic, some oncologists have growing concerns about the safety of immunotherapy because of the risk of the uncontrolled inflammatory response of patients who are infected with COVID-19 [3]. Due to the unclear impacts of immunotherapy on cancer patients who had concurrent COVID-19, oncologists are currently confronted with difficulties in the management and treatment of cancer patients [4]. Given this, the delay or cancellation of planned immunotherapy might occur, negatively affecting patients' prognosis [5].

Although most clinical trials of immunotherapy have precluded patients with active virus infection because of concerns about disease reactivation and immune-related adverse events [6], a few studies assessing the effects of implementing immunotherapy in cancer patients with active virus infection have shown that different viruses have different effects on prognosis. Regarding human immunodeficiency virus (HIV), a prior study indicated that immunotherapy was safe and feasible among patients with non-small-cell lung cancer (NSCLC) and active HIV infection, and the expression of programmed death-ligand 1 (PD-L1) was much higher in infected individuals than in their counterparts [7]. Moreover, in terms of human papillomavirus (HPV), the results of a phase 3 clinical trial focusing on unresectable or metastatic head and neck cancer showed that immunotherapy had more benefit on overall survival (OS) among PD-L1 expressors with HPV-positive tumours than their counterparts with HPV-negative tumours [8]. Similarly, Epstein–Barr virus (EBV) infection was found to be significantly associated with positive programmed death-1 (PD-1) staining in patients with locoregionally advanced nasopharyngeal carcinoma, and positive PD-1 staining was independently identified as a predictor for improved prognosis [9]. However, with regard to hepatitis B virus (HBV), a clinical trial in patients with B-cell lymphoma illustrated the risk of HBV reactivation when patients coinfected with HBV were receiving immunotherapy, which might lead to a poorer prognosis [10]. Additionally, Tapia Rico et al. [11] indicated that HBV-positive cancer patients who were treated with ICIs had the hazard of developing HBV reactivation, which could result in severe or critical immune-related adverse events.

For SARS-CoV-2, many studies have been published, while only a marginal number of prior meta-analyses have focused on the efficiency and safety of administering immunotherapy for cancer patients during the pandemic, and solid evidence is still lacking [12, 13]. Because of the lack of evidence, current clinical practice guidelines provide no clear recommendation for administering immunotherapy in cancer patients who had concurrent COVID-19. The World Health Organization (WHO) recommended continuing or modifying previous treatment according to the patient's clinical condition [14]. Similarly, the American Society of Clinical Oncology (ASCO) and the European Society for Medical Oncology (ESMO) recommended making decisions on the basis of discreet evaluation of benefits and risks for cancer patients [4, 15].

Hence, considering the points mentioned above, we conducted the present meta-analysis, for evaluating the relationship between active immunotherapy and outcomes of cancer patients who had concurrent COVID-19 infection.

## MATERIALS AND METHODS

We conducted and reported the systematic review and meta-analysis, in accordance with the Preferred Reporting Items for Systematic Reviews and Meta-Analyses (PRISMA) guidelines [16]. The protocol of the systematic review and meta-analysis has been registered in the PROSPERO database (CRD42021274069).

### Inclusion and exclusion criteria

The eligibility of the studies was assessed according to the following criteria. (1) The participants included cancer patients who were diagnosed with COVID-19 by RT–PCR and receiving active immunotherapy. (2) The intervention included immunotherapy within 30 days before the diagnosis of COVID-19. (3) The control group included cancer patients who did not receive immunotherapy within 30 days before the diagnosis of COVID-19. (4) The primary outcome was defined as mortality, and the secondary outcome was defined as the rate of severe/critical disease. The definition of severe/critical disease was in accordance with the WHO guidelines [14]. (5) All kinds of prospective and retrospective studies with extractable odds ratios (ORs), relative risks (RRs), or relevant statistics to calculate ORs and RRs were included. If the same cases from the same cohort were reported in more than one study, only the most recent study or the study reporting the most cases was included.

Studies were excluded based on the following exclusion criteria: (1) basic research, review, news, conference, guideline, editorial, comment, clinical experience, case report, and study protocol; (2) studies in which data were missing from a group of patients or data of patients receiving immunotherapy could not be separated from the whole patient group; (3) patients in whom cancer had been cured before the diagnosis of COVID-19; and (4) patients who were diagnosed with other viral pneumonias.

### Search strategy

A comprehensive search strategy was designed and performed. Studies were searched and retrieved from databases, including Embase, the Cochrane Library, Web of Science, PubMed, and the China National Knowledge Infrastructure (CNKI). The published studies were searched from 01-Dec-2019 to 01-Aug-2021. Supplementary Table 1 presents the details of the search strategy for different databases. No language limitations were imposed. Moreover, the reference lists of the studies were reviewed, to search for relevant articles.

Two independent reviewers blinded to each other performed the screening process. The titles and abstracts of retrieved records were initially screened for eligibility. Following this, the full-text screening was performed to obtain eligible studies, in accordance with the inclusion and exclusion criteria. If any discrepancy between the reviewers emerged, it was solved by discussion and arbitration.

### Data extraction

Data were extracted and collected from the included studies by two independent reviewers. Any discrepancy between the reviewers was solved by discussion and arbitration. The following datasets were extracted and collected by using a worksheet: name of the first author, publication year, country, study types (prospective or retrospective studies), total figure for participants, figure for males and females, median age or mean age, cancer types, number of patients receiving active immunotherapy, immunotherapy interval before COVID-19 diagnosis, and outcomes. The unadjusted and adjusted ORs were obtained from the articles. For adjusted ORs, adjusting variables for multivariate analyses were also extracted. If ORs were not provided, they were calculated based on original statistics, or RRs were extracted instead.

### Quality assessment

The Newcastle–Ottawa Quality Assessment Scale (NOS) was employed to conduct the quality assessment. The scale for cohort studies was defined as 3 sections, encompassing selection (4 points), comparability (2 points), and outcome (3 points). Similarly, the scale for case-control studies was defined as 3 sections, encompassing selection (4 points), comparability (2 points), and exposure (3 points). A study was rated as low quality if it scored less than 5 points [17]. Two independent reviewers, blinded to each other, evaluated the risk of bias. If any disagreements emerged in the process, they were discussed or settled by the third reviewer.

### Statistical analysis

The meta-analysis was performed by employing the generic inverse-variance method. ORs and their 95% confidence intervals (95% CIs) were calculated to compare the mortality and severe/critical disease rate between patients receiving active immunotherapy and the control patients. *P*<0.05 was considered statistically significant. As for heterogeneity analysis, the Cochran's Q test and inconsistency index (*I^2^*) were adopted, with *I^2^*>50% or *P*<0.1 deemed to indicate significant heterogeneity. We used random-effects model in the calculation of the pooled ORs and corresponding 95% CIs. Moreover, we conducted subgroup analyses according to study type, number of patients, cancer type, immunotherapy interval prior to the COVID-19 diagnosis, and number of patients receiving active immunotherapy.

We performed Egger's linear regression tests and Begg's rank correlation tests to estimate publication bias, in which *P*<0.1 indicated significant publication bias. Moreover, funnel plots were also provided to demonstrate the publication bias.

Furthermore, a sensitivity analysis was also performed by excluding an individual study each time to reflect whether any single study influenced the results.

A meta-regression was performed to investigate the effects of any potential sources of heterogeneity (study type, number of patients, number of patients receiving active immunotherapy, cancer type, and interval between immunotherapy and diagnosis of COVID-19). *P*<0.1 was considered statistically significant. The permutation test was also performed to validate the robustness of meta-regression. The data synthesis was performed by using RevMan, version 5.4 (The Nordic Cochrane Center, The Cochrane Collaboration, Denmark). Publication bias was calculated by using Stata, version 13.1 (Stata Corp, College Station, USA). Meta-regression and permutation tests were performed using the metafor package in R, version 5.1.1 (The R Foundation for Statistical Computing).

### Data availability statement

Data are available on reasonable request. All data relevant to the study are included in the article or uploaded as online Supplementary Information.

## RESULTS

### Study selection

**Figure 1:**
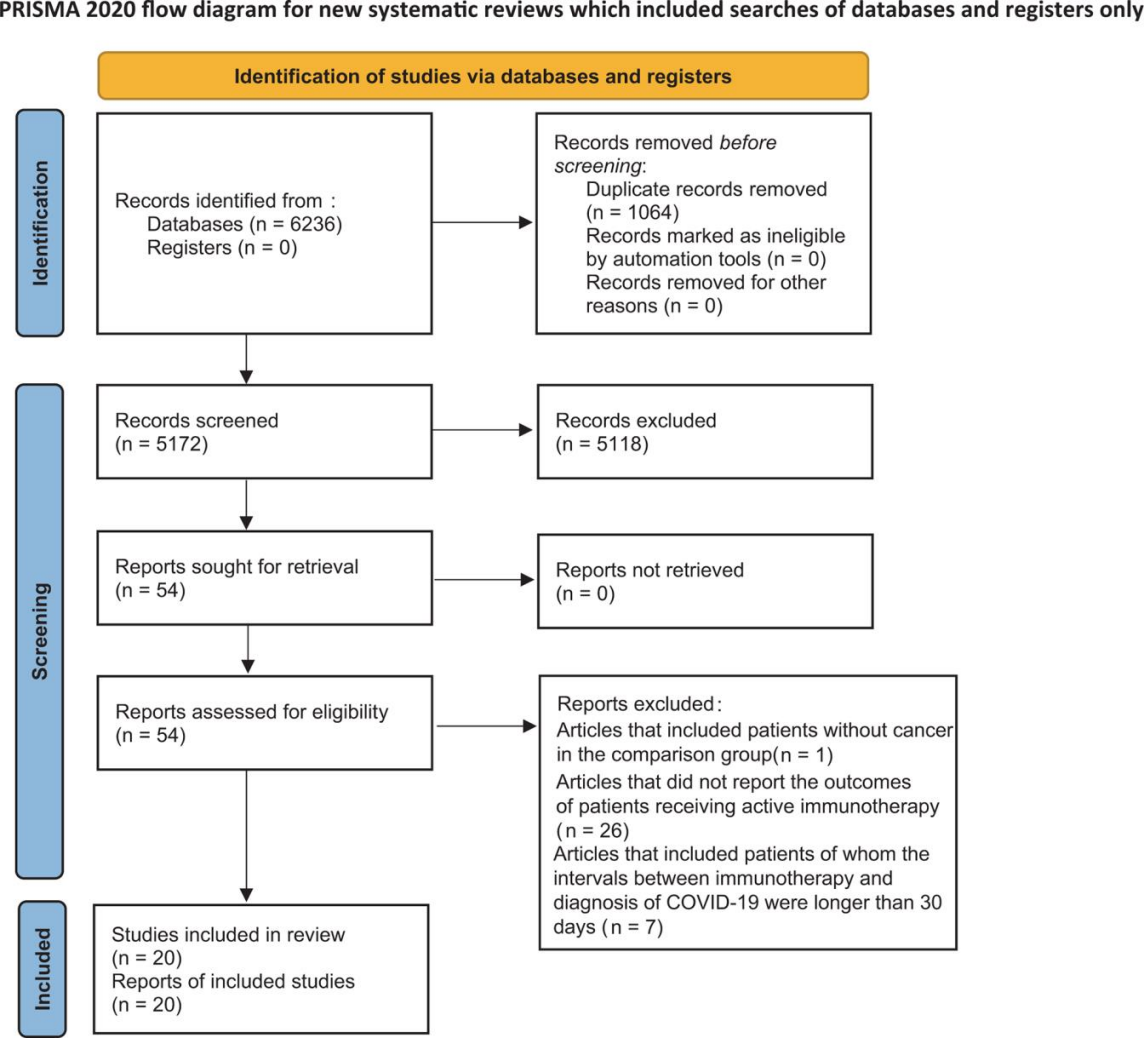
PRISMA flow diagram of study inclusion.

Figure 1 displays the flow diagram of study selection process. Overall, 6,236 articles were initially retrieved from the electronic databases, including 2,895 articles from EMBASE, 202 articles from the Cochrane Library, 1,728 articles from PubMed, 1,258 articles from Web of Science, and 153 articles from CNKI. Then, 1,064 duplicate studies were excluded, followed by the screening process. Fifty-four potentially eligible articles remained and were assessed for eligibility by full-text screening. Finally, 20 articles fully meeting the inclusion criteria were included in subsequent meta-analysis [18–37]. Supplementary Table 2 lists the articles assessed for eligibility by full-text screening, in which reasons why studies were excluded are also shown.

### Study main characteristics

Table 1 demonstrates the baseline characteristics of the included studies. Overall, five of the 20 included studies were prospective cohort studies [22, 29, 30, 35, 37]. The remainder were retrospectively designed [18–21, 23–28, 31–34, 36]. As a whole, 6,042 cancer patients diagnosed with COVID-19 were included, with 464 patients receiving active immunotherapy. Among the included 20 studies, five of them included patients with solid cancer [20, 23, 29, 33, 35], and four studies included patients with haematological malignancies [19, 30, 32, 37]. With regard to the remaining 11 studies, the included cancer types were nonspecific [18, 21, 22, 24–28, 31, 34, 36].

**Table 1:** Characteristics of the included studies.

**Study ID**	**Study type**	**Country**	**Number of patients**	**M/F^a^**	**Median age (IQR)(years)^b^**	**Cancer type**	**Interval^c^(days)**	**Number of patients^d^**
Assaad 2020	Retrospective	France	302	144/158	58.2^#^	Non-specific	30	26
Fox 2020	Retrospective	UK	55	38/17	63(23-88)	Hematological malignancies	14	9
Garassino 2020	Retrospective	International	200	141/59	68(61.8-75)	Thoracic cancer	7 (median)	34
García-Suárez 2020	Prospective	Spain	697^e^	413/277	72 (60-79)	Hematological malignancies	30	44
Jee 2020	Retrospective	US	309	119/150	NA^f^	Non-specific	35	18
Lee 2020	Prospective	UK	800^g^	449/349	69(59-76)	Non-specific	28	44
Lievre 2020	Retrospective	France	1289	795/494	67(19-100)	Solid cancer	28	62
Mehta 2020	Retrospective	US	218	127/91	69(10-92)	Non-specific	30	5
Mehta 2021	Retrospective	India	186	105/81	52(42–58.75)	Non-specific	30	11
Nakamura 2021	Retrospective	Japan	32	22/10	74.5(24–90)	Non-specific	30	3
Ozer 2021	Retrospective	US	68	37/31	72(23-91)	Non-specific	28	2
Pinato 2020	Retrospective	International	890	503/387	68^#^	Non-specific	19 (mean)	56
Provencio 2021	Prospective	Spain	447	332/115	67.1^#^	Lung cancer	NA	91
Sanchez-Pina 2020	Prospective	Spain	39	23/16	64.7^#^	Hematological malignancies	NA	3
Stroppa 2020	Retrospective	Italy	25	20/5	71.64^#^	Non-specific	NA	4
Wang 2020	Retrospective	US	58	30/28	67	Multiple myeloma	NA	32
Yang F 2020	Retrospective	China	52	28/24	63(34–98)	Solid cancer	30	1
Yang KY 2020	Retrospective	China	205	96/109	63(56–70)	Non-specific	28	4
Yarza 2020	Prospective	Spain	63	34/29	66^#^	Solid cancer	28	8
Zhang 2020	Retrospective	China	107	60/47	66(36-98)	Non-specific	30	6

^a^M means males and F means females.

^b^IQR means interquartile range.

^c^Interval of immunotherapy before diagnosis of COVID-19.

^d^Number of cancer patients receiving immunotherapy within 30 days before COVID-19 diagnosis.

^e^Data are missing for 7 patients.

^f^NA means data not available.

^g^2 patients did not identify as either male or female.

^#^Mean age.

Supplementary Table 3 mirrors the results of the quality assessment. The NOS score of the included articles ranged between 6 and 8. None of the included studies was rated as low quality.

### The effects of immunotherapy on cancer patients with COVID-19

**Figure 2:**
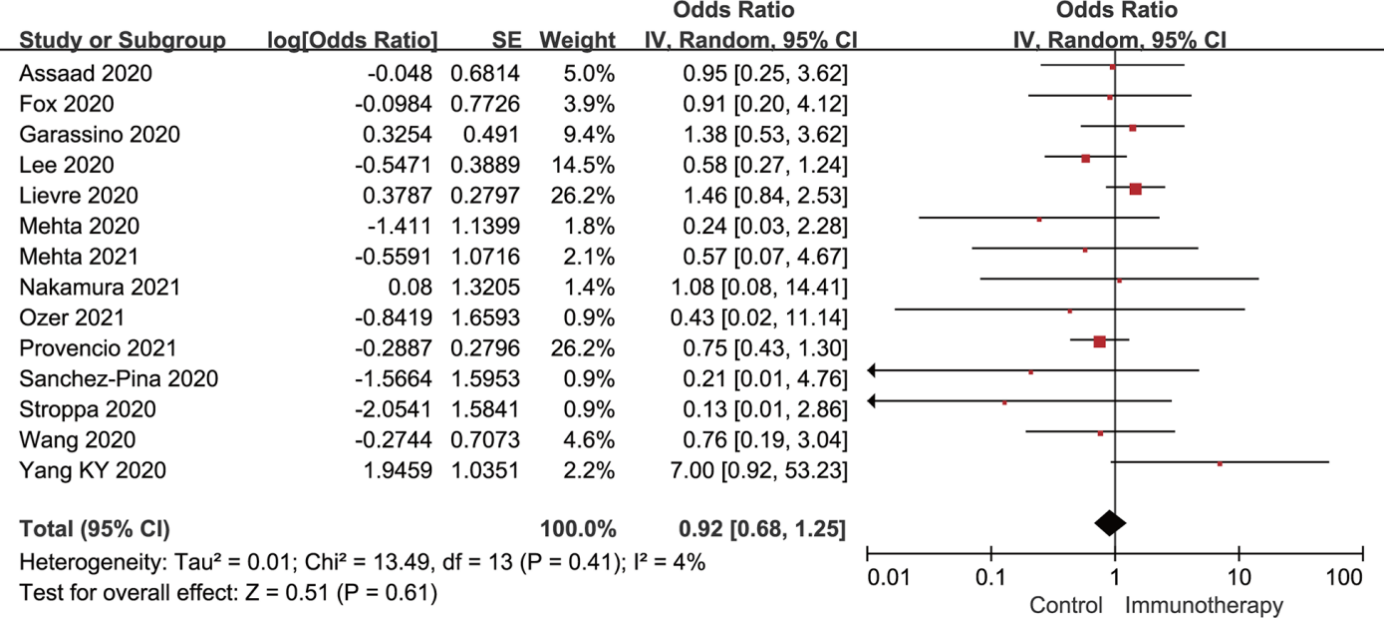
**Forest plot of the univariate analysis for the association between active immunotherapy and mortality.** CI, confidence interval; IV, inverse variance; SE, standard error.

**Figure 3:**
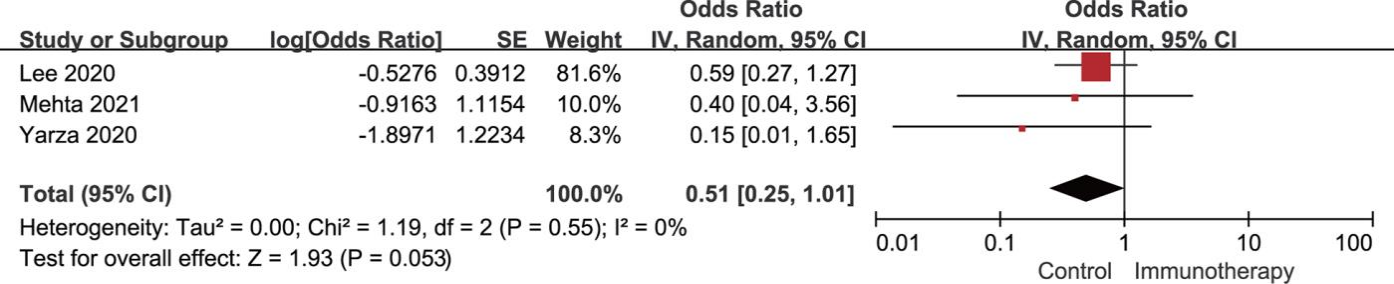
**Forest plot of the multivariate analysis for the association between active immunotherapy and mortality.** Adjusted variables for the study by Lee 2020 [22]: age, sex, and presence of comorbidities; adjusted variables for the study by Mehta 2021 [24]: age and presence of comorbidities; and adjusted variables for the study by Yarza 2020 [35]: age, sex, Eastern Cooperative Oncology Group score (ECOG), presence of metastasis, previous venous thromboembolic event (VTE), and presence of chronic obstructive pulmonary disease (COPD). CI, confidence interval; IV, inverse variance; SE, standard error.

According to the univariate analysis, the acceptance of active immunotherapy was not in relation to the increased mortality of cancer patients (OR: 0.92; 95% CI: 0.68-1.25; *P*=0.61), with no significant between-study heterogeneity found (*I* ^2^=4%; *P*=0.41). A forest plot of the unadjusted OR for the relationship between active immunotherapy and mortality of cancer patients who had concurrent COVID-19 is shown in Figure 2. Moreover, after adjusting for confounders, the adjusted OR was 0.51, with borderline significance (95% CI: 0.25-1.01; *P*=0.053), as shown in Figure 3. No significant heterogeneity was observed between the studies providing adjusted results of mortality (*I* ^2^=0%; *P*=0.55).

Subgroup analyses for mortality of cancer patients co-diagnosed with COVID-19 were performed, of which the unadjusted ORs are mirrored in Table 2. As reflected in the subgroup analyses, no variables investigated were related to significant ORs (*P*>0.05), revealing that active immunotherapy was not in relation to increased mortality of cancer patients, regardless of confounders.

**Table 2:** Meta-analyses and subgroup analyses for mortality.

**Patients receiving immunotherapy vs. control patients**	**N of studies**	**Pooled OR (95%CI) ^a^**	***I* ^2^ (%) ^b^**	***P***	***P* for interaction**
Overall	14	0.92 (0.68, 1.25)	4%	0.61	
Study type					
Prospective	3	0.67 (0.43, 1.04)	0%	0.07	0.054
Retrospective	11	1.19 (0.81, 1.73)	0%	0.38	
Number of patients					
<100	6	0.64 (0.28, 1.49)	0%	0.30	0.39
>100	8	0.97 (0.63, 1.49)	34%	0.90	
Cancer type					
Hematological malignancies	3	0.72 (0.27, 1.91)	0%	0.51	0.48
Solid tumor	3	1.10 (0.69, 1.76)	36%	0.68	
Non-specific cancer	8	0.72 (0.39, 1.33)	8%	0.29	
Immunotherapy interval before the COVID-19 diagnosis (days)					
>20	8	0.98 (0.56, 1.69)	25%	0.94	0.44
<20	2	1.23 (0.54, 2.76)	0%	0.62	
Number of patients receiving active immunotherapy					
<10	7	0.75 (0.27, 2.08)	22%	0.58	0.66
>10	7	0.95 (0.70, 1.28)	0%	0.73	

^a^Calculated by using the random-effect model.

^b^*I* ^2^ means the inconsistency across studies.

**Figure 4:**
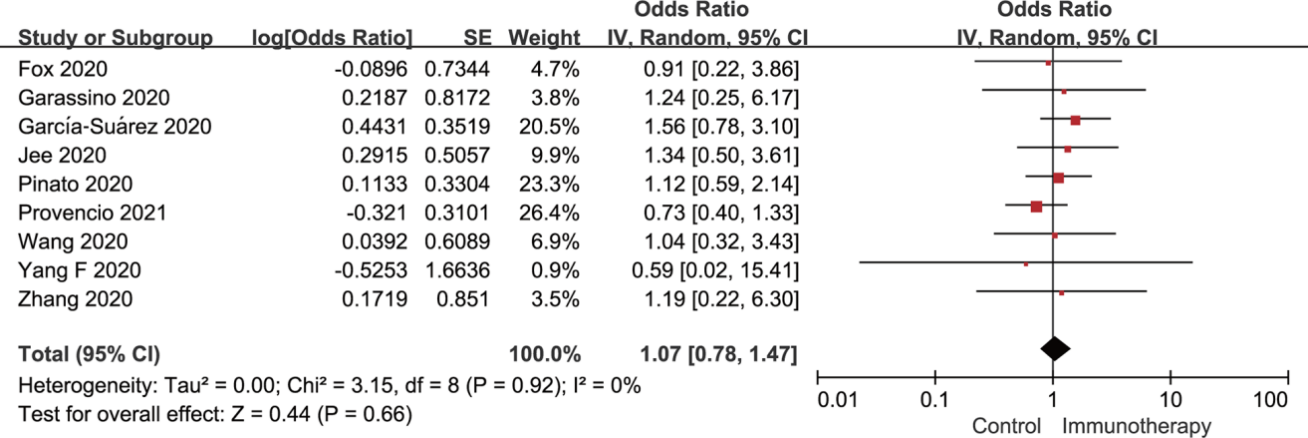
**Forest plot of the univariate analysis for the association between active immunotherapy and severe/critical disease rate.** CI, confidence interval; IV, inverse variance; SE, standard error.

For the meta-analysis of the severe/critical disease rate, the definition of WHO guidelines was employed [14]. Figure 4 shows a forest plot of the OR for the relationship between active immunotherapy and the rate of severe/critical disease in cancer patients who had concurrent COVID-19. According to the univariate analysis, active immunotherapy was not in relation to increased risk of severe/critical disease of cancer patients (OR: 1.07; 95% CI: 0.78-1.47; *P*=0.66), with nonsignificant between-study heterogeneity found (*I* ^2^=0%; *P*=0.92).

Supplementary Tables 4, 5 mirrors the results of meta-regression. None of the tested covariates could yield heterogeneity, with *P*>0.1. Permutation tests showed that the results of meta-regression were reliable.

### Sensitivity analysis and publication bias

In order to evaluate the stability of the present results, sensitivity analyses were performed by excluding an individual study each time to reflect whether any single study influenced the results. The results of the sensitivity analysis indicated that the pooled ORs were not significantly influenced by excluding any single study. Furthermore, Egger's linear regression tests and Begg's rank correlation tests were conducted through which nonsignificant publication bias was shown in the studies regarding mortality and the studies regarding severe/critical disease, as illuminated in Supplementary Figures 1–4. Supplementary Figures 5, 6 are funnel plots of included studies.

## DISCUSSION

The present study indicated that active immunotherapy was not associated with increased mortality or rate of severe/critical disease in cancer patients who had concurrent COVID-19 infection. Some discrepancies were found between the results yielded by the present study and those yielded by the previous studies, and those studies must be updated. In the results published by Liu et al. [13], immunotherapy was found to have a tendency of increasing the risk of mortality (RR: 1.20; 95% CI: 0.68-2.13) and severe/critical disease (RR: 1.24; 95% CI: 0.94-1.63). Moreover, Liu et al. [13] indicated that immunotherapy had higher risk compared with other anticancer treatments. The variation might partially derive from the inclusion of newly published studies in the present meta-analysis. The research of Liu et al. [13] was conducted in the comparatively earlier period of the pandemic, and the number of published studies regarding COVID-19 and cancer was limited. Furthermore, the results reported by Liu et al. [13] were all unadjusted; however, the accuracy and reliability of these unadjusted results might be affected by a series of confounding variables (e.g., age, sex, presence of comorbidities, smoking status, presence of metastasis), particularly in retrospective studies. In the study of Yekeduz et al. [12], immunotherapy was detected to have a potential risk of increasing mortality (OR: 1.12; 95% CI: 0.60-2.08) and the rate of severe/critical disease (RR: 1.60; 95% CI: 0.72-3.52). The discrepancy might be due to the inappropriate inclusion of the study published by Dai et al. [38], in which the comparison group was patients without cancer, resulting in significantly higher heterogeneity (*I^2^*>50%) [12].

Granted, some inconsistencies were observed, and there were still some studies supporting the present results. A previous study involving 522 patients with concurrent COVID-19 demonstrated that the number of T cells were drastically diminished in COVID-19 patients. Moreover, T cell exhaustion was also observed, concomitant with the higher expression of PD-1 and increased serum IL-6 and IL-10 in patients who were infected by COVID-19 [39]. In light of this immune response, immunotherapy administration might be conducive to patients with COVID-19 because it activates exhausted T cells by blocking PD-1/PD-L1 or CTLA-4 [40]. Furthermore, Yekeduz et al. [12] indicated that the use of immunotherapy, especially ICIs, was safe in cancer patients during the pandemic, which was in line with our results.

The present study has strengths, including comprehensive inclusion and a multivariate analysis. The figure for studies regarding immunotherapy included in this meta-analysis surpassed those in prior studies. In addition, the low publication bias and between-study heterogeneity contributed to more reliable and conservative results as well as higher quality of evidence. Moreover, in the special period of the COVID-19 outbreak, one of the major concerns is the safety of using immunotherapy to treat cancer patients, by virtue of the immune-related adverse events, which can probably lead to worsening prognosis of cancer patients who had concurrent COVID-19 infection [3]. However, the results derived from the present meta-analysis indicated that administering immunotherapy in cancer patients during the pandemic of COVID-19 was not associated with risk of death and severe/critical COVID-19. This provides more evidence for oncologists when managing cancer patients in the special era.

Some limitations should be addressed in the present research. First, the control groups were observed to be inconsistent in the included studies. Fifteen studies included cancer patients not receiving any active anticancer treatment in control groups, while the remaining five studies included cancer patients without active immunotherapy. Considering this, we conducted sensitivity analyses, of which the results showed that the exclusion of these five studies did not significantly alter the pooled results. Second, the definitions of severe/critical diseases were not totally consistent among included studies. This inconsistency might introduce the risk of bias to the present results. Third, due to the lack of adjusted results, we did not conduct a multivariate analysis of the severe/critical disease rate. The results of the univariate analysis may not mirror the real effects of immunotherapy on cancer patients as many confounders can affect the prognosis of cancer and COVID-19. Fourth, a majority of the included studies were retrospective. Fifth, some studies did not include sufficient patients on immunotherapy, which might introduce bias to the results. Although we did not find significant statistical heterogeneity or publication bias, the results yielded by the present meta-analysis should be discreetly interpreted in clinical practice, in combination with the assessment of specific patient conditions.

## CONCLUSIONS

Accepting immunotherapy within 30 days before the diagnosis of COVID-19 was not significantly associated with a higher risk of mortality or severe/critical disease of infected cancer patients. Due to the limitations of the present study, the conclusions should be interpreted with discretion, and further prospectively designed studies with large sample sizes are required to evaluate the present results.

## Supplementary Material

Supplementary Figures

Supplementary Tables
