# Supplementary material for: Association of active immunotherapy with outcomes in cancer patients with COVID-19: a systematic review and meta-analysis
Source: Aging (Albany NY). 2022 Mar 10;14(5):2062–80. doi: 10.18632/aging.203945 (PMC8954969; doi:10.18632/aging.203945)
Supplement: Supplementary Figures [file aging-14-203945-s001.pdf]

SUPPLEMENTARY FIGURES

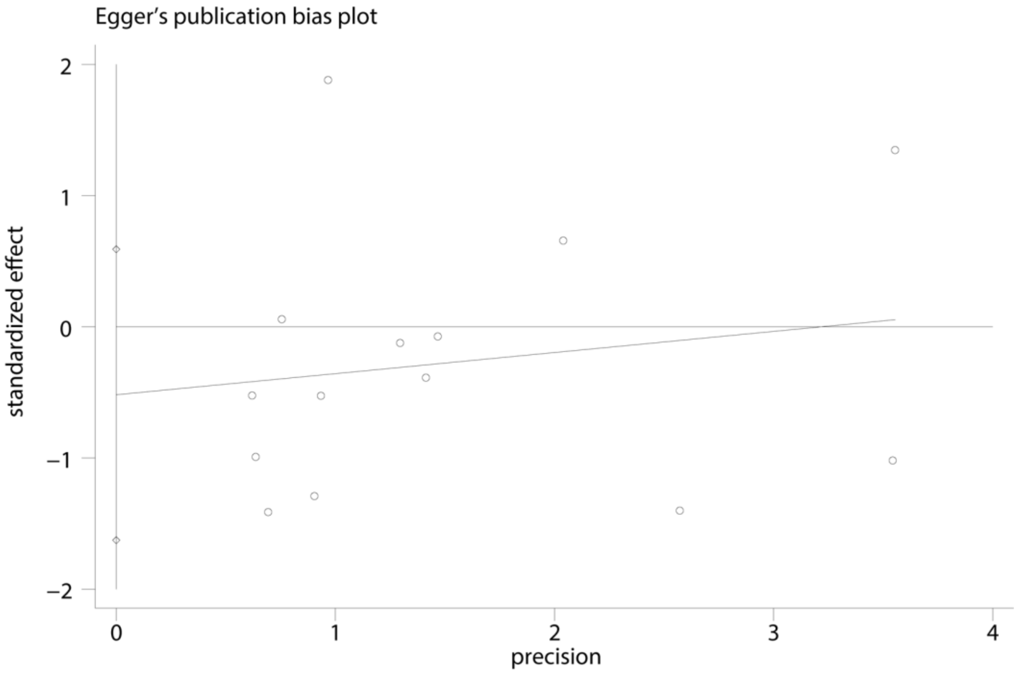

P=0.329

Supplementary Figure 1. Publication bias of studies regarding mortality (Egger's linear regression test).

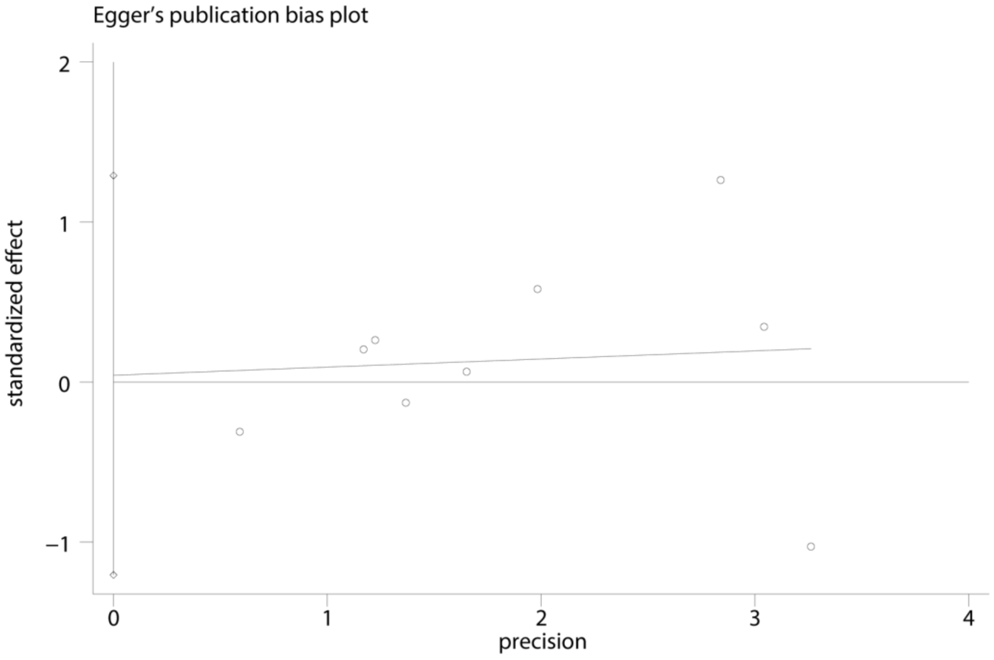

P=0.938

Supplementary Figure 2. Publication bias of studies regarding severe/critical disease (Egger's linear regression test).

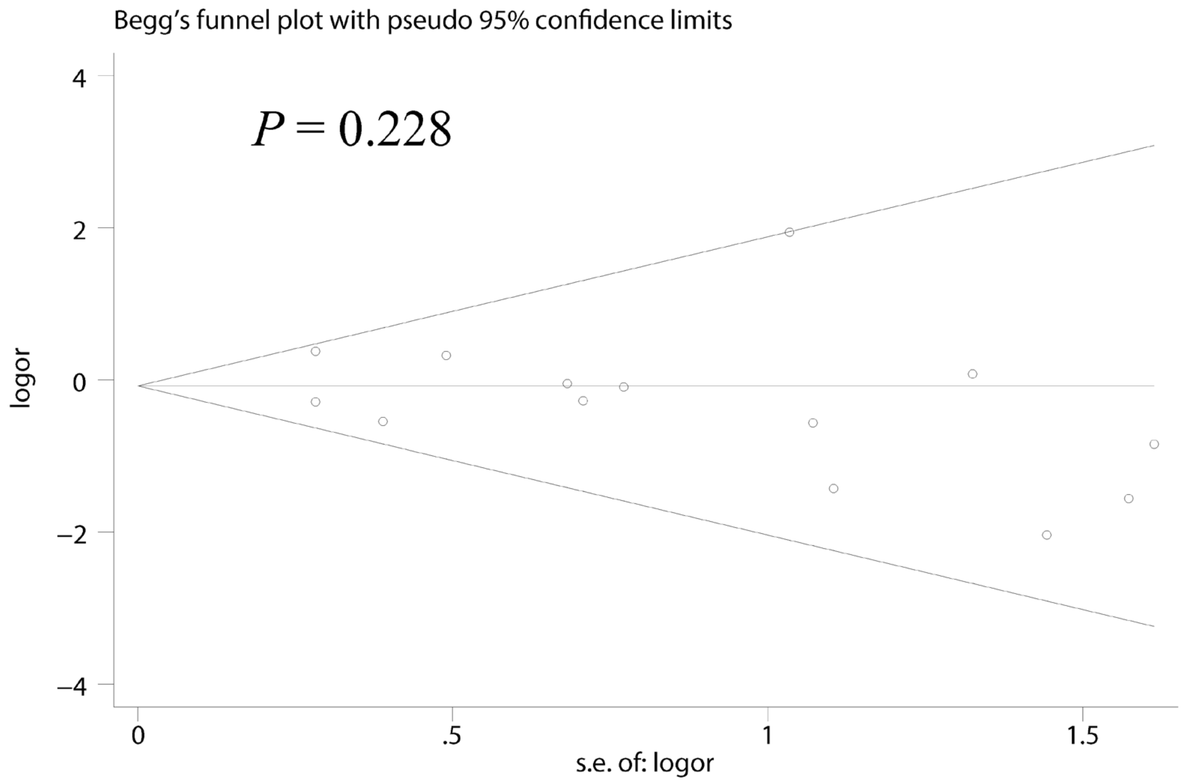

Supplementary Figure 3. Publication bias of studies regarding mortality (Begg's rank correlation test).

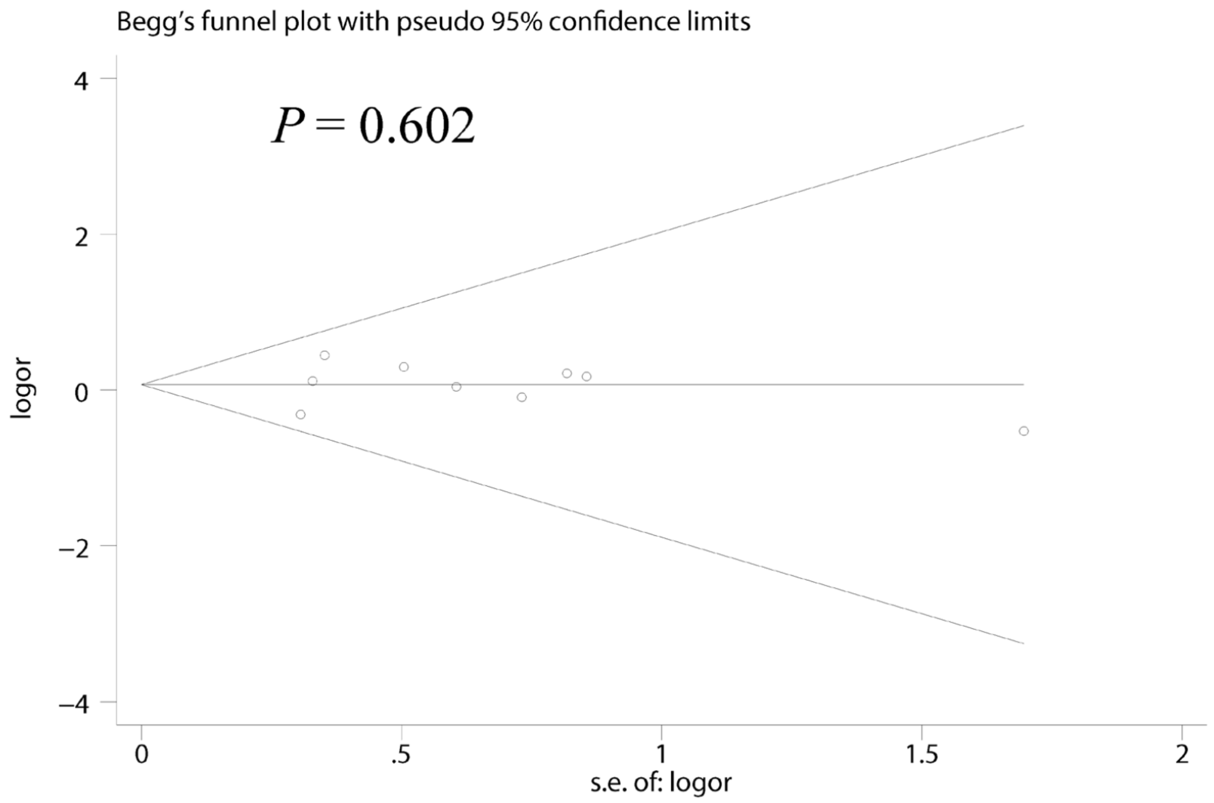

Supplementary Figure 4. Publication bias of studies regarding severe/critical disease (Begg's rank correlation test).

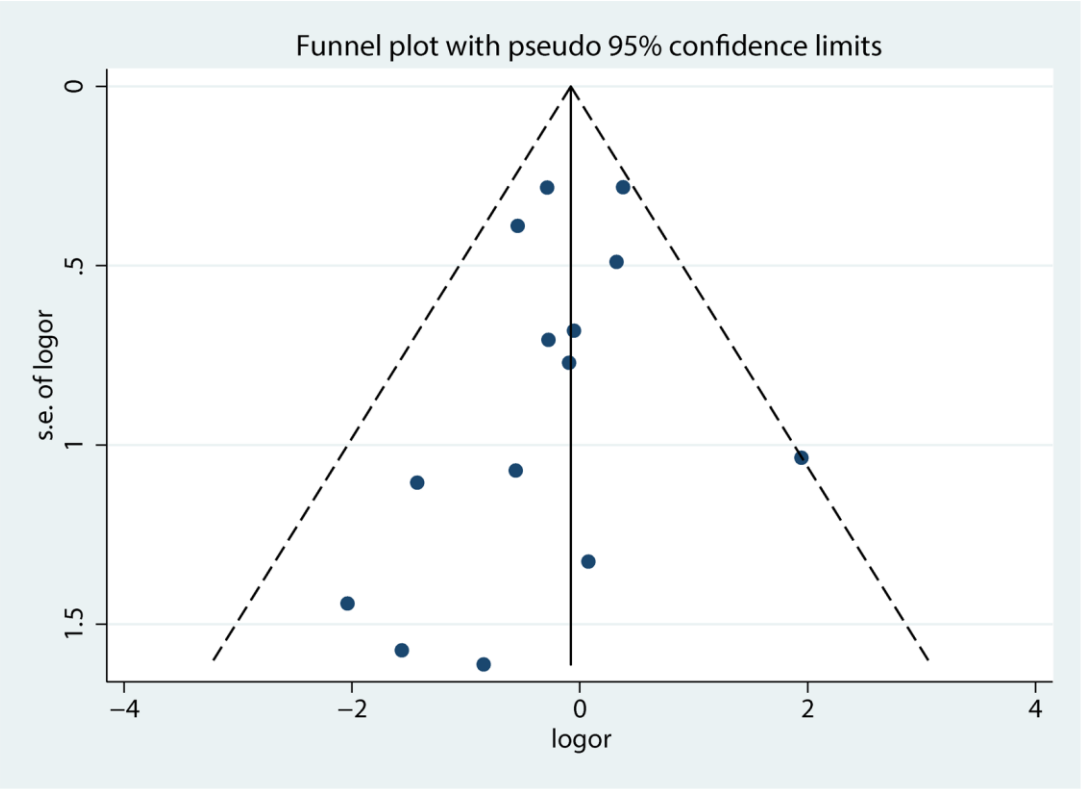

Supplementary Figure 5. Publication bias of studies regarding mortality (funnel plot).

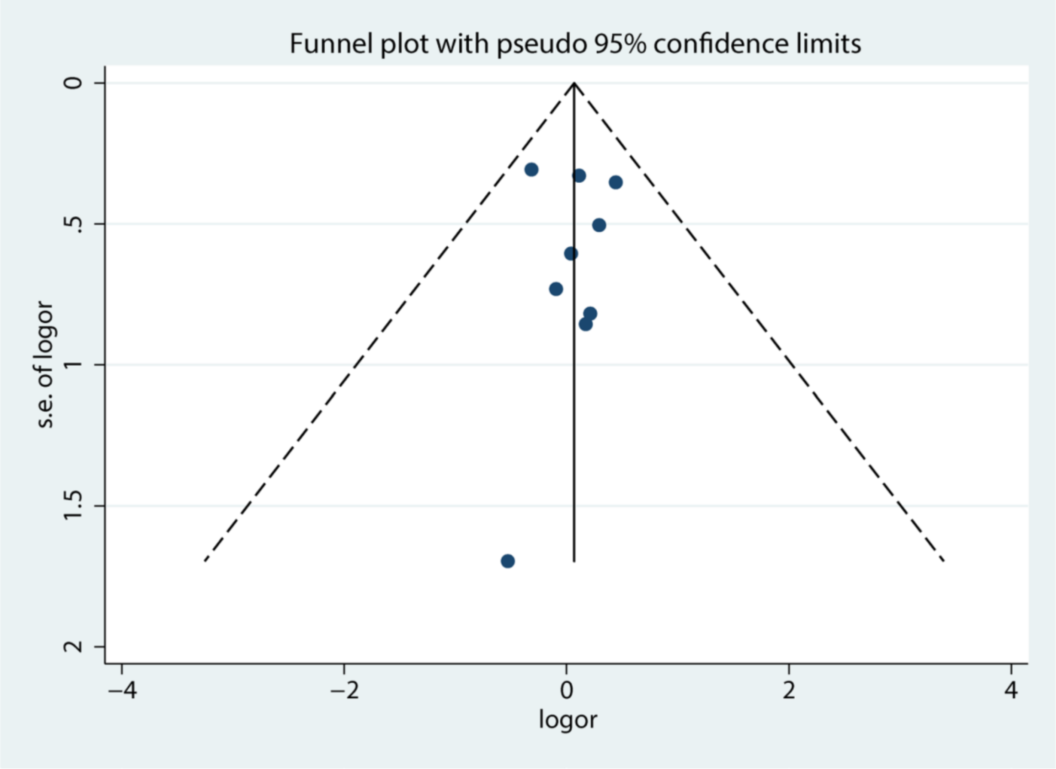

Supplementary Figure 6. Publication bias of studies regarding severe/critical disease (funnel plot).
