# Supplementary material for: Association of active immunotherapy with outcomes in cancer patients with COVID-19: a systematic review and meta-analysis
Source: Aging (Albany NY). 2022 Mar 10;14(5):2062–80. doi: 10.18632/aging.203945 (PMC8954969; doi:10.18632/aging.203945)
Supplement: Supplementary Tables [file aging-14-203945-s002.pdf]

SUPPLEMENTARY TABLES

Supplementary Table 1. Search strategy.

	**Embase**	
1	exp immunotherapy/	58400
2	(immunotherapy or immunotherapies or immunotherapeutic or immunotherapeutics or immuno* or immune* or immunothera* or ICI or ICIs or CPI or immune-checkpoint inhibitor or immune checkpoint inhibitor or (immune adj2 checkpoint) or immune-checkpoint blockade or immune checkpoint blockade or immune?checkpoint* or nivolumab or pembrolizumab or atezolizumab or avelumab or durvalumab or ipilimumab or PD-1 or PD-L1 or (PD adj3 immunotherapy) or CTLA-4 or CTLA?4).mp.	703128
3	exp cancer/	641207
4	(tumor or carcinoma or cancer or malignant or malignancy or malignan* or neoplasia or neoplasm or neoplastic or neopla* or carcinoma or carcinomatous or carcino* or adenocarcinoma or metastic or metastases or metastasis or oncology or oncological or hematology or hematolog* or haematolog* or leukemia or lymphoma or myeloma).mp.	993990
5	exp COVID-19/	145801
6	(COVID-19 or SARS-CoV-2 or Novel coronavirus or Wuhan coronavirus or 2019 coronavirus or COVID or pandemic).mp.	180887
7	(1 or 2) and (3 or 4) and (5 or 6)	3238
8	exp humans/ not animals.sh.	3415527
9	7 and 8	2895
	**PubMed**	
1	immunotherapy	40,439
2	immunotherapy or immunotherapies or immunotherapeutic or immunotherapeutics or ICI or ICIs or CPI or immune-checkpoint inhibitor or immune checkpoint inhibitor or immune-checkpoint blockade or immune checkpoint blockade or nivolumab or pembrolizumab or atezolizumab or avelumab or durvalumab or ipilimumab or PD-1 or PD-L1 or CTLA-4	52,369
3	cancer	414,013
4	tumor or carcinoma or cancer or malignant or malignancy or neoplasia or neoplasm or neoplastic or carcinoma or carcinomatous or adenocarcinoma or metastic or metastases or metastasis or oncology or oncological or hematology or leukemia or lymphoma or myeloma	738,253
5	COVID-19	166,753
6	COVID-19 or SARS-CoV-2 or Novel coronavirus or Wuhan coronavirus or 2019 coronavirus or COVID or pandemic	171,755
7	(1 or 2) and (3 or 4) and (5 or 6)	3785
8	animals[Title/Abstract]	57,424
9	7 not 8	1728
	**Web of science**	
1	TS=(immunotherapy)	35,556
2	TS=(immunotherapy or immunotherapies or immunotherapeutic or immunotherapeutics or immuno* or immune* or immunothera* or ICI or ices or CPI or immune-checkpoint inhibitor or immune checkpoint inhibitor or immune-checkpoint blockade or immune checkpoint blockade or immune?checkpoint* or nivolumab or pembrolizumab or atezolizumab or avelumab or durvalumab or ipilimumab or PD-1 or PD-L1 or CTLA-4)	602,701
3	TS=(cancer)	447,446
4	TS=(tumor or carcinoma or cancer or malignant or malignancy or malignan* or neoplasia or neoplasm or neoplastic or neopla* or carcinoma or carcinomatous or carcino* or adenocarcinoma or metastic or metastases or metastasis or oncology or oncological or hematology or hematolog* or haematolog* or leukemia or lymphoma or myeloma)	765,618
5	TS=(COVID-19)	191,781

6	TS=(COVID-19 or SARS-CoV-2 or Novel coronavirus or Wuhan coronavirus or 2019 coronavirus or COVID or pandemic)	212,159
7	(1 or 2) and (3 or 4) and (5 or 6)	4196
8	TS=(animals)	1,440,043
9	7 not 8	1258
	**EBM reviews – cochrane central register of controlled trials**	
1	exp immunotherapy/	671
2	(immunotherapy or immunotherapies or immunotherapeutic or immunotherapeutics or immuno* or immune* or immunothera* or ICI or ICIs or CPI or immune-checkpoint inhibitor or immune checkpoint inhibitor or (immune adj2 checkpoint) or immune-checkpoint blockade or immune checkpoint blockade or immune?checkpoint* or nivolumab or pembrolizumab or atezolizumab or avelumab or durvalumab or ipilimumab or PD-1 or PD-L1 or (PD adj3 immunotherapy) or CTLA-4 or CTLA?4).mp.	22574
3	exp neoplasms/	9971
4	(tumor or carcinoma or cancer or malignant or malignancy or malignan* or neoplasia or neoplasm or neoplastic or neopla* or carcinoma or carcinomatous or carcino* or adenocarcinoma or metastic or metastases or metastasis or oncology or oncological or hematology or hematolog* or haematolog* or leukemia or lymphoma or myeloma).mp.	42082
5	(COVID-19 or SARS-CoV-2 or Novel coronavirus or Wuhan coronavirus or 2019 coronavirus or COVID or pandemic).mp.	7342
6	(1 or 2) and (3 or 4) and 5	202
	**China national knowledge infrastructure**	
1	immunotherapy	26,785
2	immunotherapy or immune-checkpoint inhibitor or PD-1 or PD-L1 or CTLA-4	39,239
3	cancer	368,546
4	cancer or tumor or blood or leukemia or lymphoma	741,583
5	COVID-19	25,561
6	COVID-19 or SARS-CoV-2	36,920
7	(1 or 2) and (3 or 4) and (5 or 6)	153

Supplementary Table 2. List of articles assessed for eligibility.

ID	DOI	Include	Reason of exclusion
Assaad 2020	10.1016/j.ejca.2020.05.028	√	
Fox 2020	10.1111/bjh.17027	√	
Garassino 2020	10.1016/S1470-2045(20)30314-4	√	
García-Suárez 2020	10.1186/s13045-020-00970-7	√	
Jee 2020	10.1200/JCO.20.01307	√	
Lee 2020	10.1016/S0140-6736(20)31173-9	√	
Lievre 2020	10.1016/j.ejca.2020.09.035	√	
Mehta 2020	10.1158/2159-8290.CD-20-0516	√	
Mehta 2021	10.7717/peerj.10599	√	
Nakamura 2021	10.1007/s10147-020-01837-0	√	
Ozer 2021	10.1016/j.ctarc.2021.100418	√	
Pinato 2020	10.1158/2159-8290.CD-20-0773	√	
Provencio 2021	10.1016/j.lungcan.2021.05.014	√	
Sanchez-Pina 2020	10.1111/ejh.13493	√	
Stroppa 2020	10.2217/fon-2020-0369	√	
Wang 2020	10.1186/s13045-020-00934-x	√	
Yang F 2020	10.1002/jmv.25972	√	
Yang KY 2020	10.1016/S1470-2045(20)30310-7	√	
Yarza 2020	10.1016/j.ejca.2020.06.001	√	
Zhang 2020	10.1002/cncr.33042	√	
Dai 2020	10.1158/2159-8290.CD-20-0422		√ (included patients without cancer in the comparison group)
Fu 2021	10.1002/cncr.33657		√ (intervals between immunotherapy and diagnosis of COVID-19 > 30d)
Lara 2020	10.1002/cncr.33084,		√ (intervals between immunotherapy and diagnosis of COVID-19 > 30d)
Luo 2020	10.1016/j.annonc.2020.06.007		√ (intervals between immunotherapy and diagnosis of COVID-19 > 30d)
Martin 2021	10.1002/onco.13831		√ (intervals between immunotherapy and diagnosis of COVID-19 > 30d)
Robilotti 2020	10.1038/s41591-020-0979-0		√ (intervals between immunotherapy and diagnosis of COVID-19 > 30d)
Russell 2020	10.3389/fonc.2020.01279		√ (intervals between immunotherapy and diagnosis of COVID-19 > 30d)
Song 2021	10.1002/cncr.		√ (intervals between immunotherapy and diagnosis of COVID-19 > 30d)
Ali 2020	10.1016/j.hemonc.2020.12.001		√ (did not report the outcomes of patients receiving active immunotherapy)
Booth 2020	10.1111/EJH.13469		√ (did not report the outcomes of patients receiving active immunotherapy)
Caffo 2020	10.1016/j.ejca.2020.09.018		√ (did not report the outcomes of patients receiving active immunotherapy)
Cattaneo 2020	10.1002/cncr.33160		√ (did not report the outcomes of patients receiving active immunotherapy)
Di Cosimo 2021	10.3390/cancers13061324		√ (did not report the outcomes of patients receiving active immunotherapy)
Guarneri 2021	10.1016/j.ejca.2021.01.021		√ (did not report the outcomes of patients receiving active immunotherapy)
Kuderer 2020	10.1016/S0140-6736(20)31187-9		√ (did not report the outcomes of patients receiving active immunotherapy)
Li 2020	10.1038/s41375-020-0986-7		√ (did not report the outcomes of patients receiving active immunotherapy)
Liang 2021	10.1007/s11684-021-0845-6		√ (did not report the outcomes of patients receiving active immunotherapy)
Liu 2020	10.1136/jitc-2020-001314		√ (did not report the outcomes of patients receiving active immunotherapy)
Liu 2021	10.7150/jca.54205		√ (did not report the outcomes of patients receiving active immunotherapy)
Ma 2020	10.1016/j.jinf.2020.04.006		√ (did not report the outcomes of patients receiving active immunotherapy)
Martín-Moro 2020	10.1111/bjh.16801		√ (did not report the outcomes of patients receiving active immunotherapy)
Mato 2020	10.1182/blood.2020006965.		√ (did not report the outcomes of patients receiving active immunotherapy)
Morais 2021	10.1002/ijc.33532		√ (did not report the outcomes of patients receiving active immunotherapy)
Nicole 2020	10.1016/S0140-6736(20)31187-9		√ (did not report the outcomes of patients receiving active immunotherapy)
Rogado 2020	10.1007/s12094-020-02381-z		√ (did not report the outcomes of patients receiving active immunotherapy)
Scarfò 2020	10.1038/s41375-020-0959-x		√ (did not report the outcomes of patients receiving active immunotherapy)
Tian 2020	10.1016/S1470-2045(20)30309-0		√ (did not report the outcomes of patients receiving active immunotherapy)
Vuagnat 2020	10.1186/s13058-020-01293-8		√ (did not report the outcomes of patients receiving active immunotherapy)
Wei 2021	10.1016/j.breast.2021.06.006		√ (did not report the outcomes of patients receiving active immunotherapy)
Zhang 2020	10.1016/j.annonc.2020.03.296		√ (did not report the outcomes of patients receiving active immunotherapy)

Argenziano 2020	10.1136/bmj.m1996	√ (did not report the outcomes of patients receiving active immunotherapy)
Feng 2020	10.1164/rccm.202002-0445OC	√ (did not report the outcomes of patients receiving active immunotherapy)
Gill 2021	10.1371/journal.pone.0248498	√ (did not report the outcomes of patients receiving active immunotherapy)
Huang 2020	10.1016/S0140-6736(20)30183-5	√ (did not report the outcomes of patients receiving active immunotherapy)

Supplementary Table 3. Quality assessment of included studies.

ID	Selection	Comparability	Exposure/Outcome	Score
Assaad 2020	**	*	***	6
Fox 2020	**	*	***	6
Garassino 2020	**	**	***	7
García-Suárez 2020	***	*	***	7
Jee 2020	**	*	***	6
Lee 2020#	***	*	***	7
Lievre 2020	***	*	***	7
Mehta 2020	**	*	***	6
Mehta 2021	**	**	***	7
Nakamura 2021	**	**	***	7
Ozer 2021	***	*	***	7
Pinato 2020	**	*	***	6
Provencio 2021[#]	***	**	***	8
Sanchez-Pina 2020	**	*	***	6
Stroppa 2020	**	*	***	6
Wang 2020	**	*	***	6
Yang F 2020	**	*	***	6
Yang KY 2020	***	**	***	8
Yarza 2020#	****	*	***	8
Zhang 2020	***	*	***	7

[#] Quality assessment performed by using Newcastle-Ottawa scale (NOS) for cohort studies. Remainder assessed by using the NOS for case-control studies.

Supplementary Table 4. Meta-regression of studies regarding mortality.[a]

Covariate	Estimate	Standard error	t-value	p-value	Estimate LCI[b]	Estimate UCI[c]
Study type	-0.6473	0.4449	-1.4547	0.1838	-1.6733	0.3788
Number of patients	0.0002	0.0004	0.551	0.5967	-0.0007	0.0012
Cancer type	-0.0759	0.427	-0.1777	0.8634	-1.0605	0.9088
Immunotherapy interval before COVID-19 diagnosis	-0.0183	0.0273	-0.6698	0.5218	-0.0811	0.0446
Number of patients receiving active immunotherapy	0.0061	0.0117	0.5262	0.613	-0.0208	0.033

[a]The robustness of meta-regression is validated by permutation test.
[b]LCI means lower bound of 95% confidence interval.
[c]UCI means upper bound of 95% confidence interval.

Supplementary Table 5. Meta-regression of studies regarding severe/critical disease.[a]

Covariate	Estimate	Standard error	*t*-value	*p*-value	Estimate LCI[b]	Estimate UCI[c]
Study type	0.2028	0.2288	0.8864	0.4407	-0.5253	0.9309
Number of patients	0.0006	0.0003	1.9959	0.1399	-0.0004	0.0015
Cancer type	-0.0385	0.1411	-0.2727	0.8028	-0.4876	0.4106
Immunotherapy interval before COVID-19 diagnosis	0.0025	0.0117	0.2107	0.8466	-0.0347	0.0396
Number of patients receiving active immunotherapy	-0.0103	0.0055	-1.8596	0.1599	-0.0278	0.0073

[a]The robustness of meta-regression is validated by permutation test.
[b]LCI means lower bound of 95% confidence interval.
[c]UCI means upper bound of 95% confidence interval.
